# Breast Cancer Detection and Analytics Using Hybrid CNN and Extreme Learning Machine

**DOI:** 10.3390/jpm14080792

**Published:** 2024-07-26

**Authors:** Vidhushavarshini Sureshkumar, Rubesh Sharma Navani Prasad, Sathiyabhama Balasubramaniam, Dhayanithi Jagannathan, Jayanthi Daniel, Seshathiri Dhanasekaran

**Affiliations:** 1 Department of Computer Science and Engineering, SRM Institute of Science and Technology, Vadapalani, Chennai 600026, India; 2 Department of Community Medicine, Government Mohan Kumaramangalam Medical College, Salem 636030, India; dr.rubesh.smc@gmail.com; 3 Department of Computer Science and Engineering, Sona College of Technology, Salem 636005, India; sathiyabhama@sonatech.ac.in (S.B.); dhaya.j@sonatech.ac.in (D.J.); 4 Department of Electronics and Communication Engineering, Rajalakshmi Engineering College, Chennai 602105, India; jayanthivlsi@gmail.com; 5 Department of Computer Science, UiT The Arctic University of Norway, 9037 Tromsø, Norway

**Keywords:** computer-aided diagnosis (CAD), mammogram, pectoral muscle removal, breast cancer, convoluted neural networks, extreme learning machine

## Abstract

Early detection of breast cancer is essential for increasing survival rates, as it is one of the primary causes of death for women globally. Mammograms are extensively used by physicians for diagnosis, but selecting appropriate algorithms for image enhancement, segmentation, feature extraction, and classification remains a significant research challenge. This paper presents a computer-aided diagnosis (CAD)-based hybrid model combining convolutional neural networks (CNN) with a pruned ensembled extreme learning machine (HCPELM) to enhance breast cancer detection, segmentation, feature extraction, and classification. The model employs the rectified linear unit (ReLU) activation function to enhance data analytics after removing artifacts and pectoral muscles, and the HCPELM hybridized with the CNN model improves feature extraction. The hybrid elements are convolutional and fully connected layers. Convolutional layers extract spatial features like edges, textures, and more complex features in deeper layers. The fully connected layers take these features and combine them in a non-linear manner to perform the final classification. ELM performs classification and recognition tasks, aiming for state-of-the-art performance. This hybrid classifier is used for transfer learning by freezing certain layers and modifying the architecture to reduce parameters, easing cancer detection. The HCPELM classifier was trained using the MIAS database and evaluated against benchmark methods. It achieved a breast image recognition accuracy of 86%, outperforming benchmark deep learning models. HCPELM is demonstrating superior performance in early detection and diagnosis, thus aiding healthcare practitioners in breast cancer diagnosis.

## 1. Introduction

Recently, deep-learning-based approaches have been utilized in medical image processing. Many societies, especially in underdeveloped nations like India, do not routinely promote breast cancer awareness or encourage routine check-ups. Medical imaging technologies have been used mostly for screening purposes for the past few decades. The advantages of medical imaging technology, however, are contingent upon radiologists’ training and proficiency in picture interpretation. Utilizing machine learning techniques in conjunction with CAD methodologies has produced encouraging outcomes, in addition to medical imaging technology [1].

Machine learning (ML) requires massive datasets to be trained; on some occasions the results are biased, and quality is at stake. Based on the data dynamics, there may be a requirement to generate new data, and ML algorithms will not be able to do it. ML algorithms are highly susceptible to errors, which may go undetected for a long time and sometimes go unnoticed entirely. ML algorithms take more time to learn and require massive resources, with the added problem of a lack of interpretability of the results. Techniques for deep learning (DL) have advanced quickly in recent years to overcome ML’s limitations and resolve challenging issues. To extract features, deep convolutional neural networks (DCNNs) use a structure that consists of convolutional layers, pooling layers, and fully connected layers. Features are categorized and downsampled in the optimization process [1]. Local features including colors, corners, endpoints, and orientated edges are gathered in the shallow layers of convolutional layers. As the layer descends, these local features in the shallow layers integrate into larger structural elements such as ellipses, circles, certain other shapes, or patterns. These structure or pattern features then make up the high-level semantic representations that explain the feature abstraction for every category. To decrease the dimensionality of the features collected using convoluted layers, feature down-sampling is carried out in pooling layers using either average pooling or max-pooling layers [2]. However, in fully connected layers, it functions as a classifier known as multilayer perceptron (MLP) by using the characteristics that were derived from the convolutional layers as inputs. Relationships between such semantic attributes communicate the co-occurrence characteristics of patterns or objects.

### 1.1. About Breast Cancer

Uncontrolled cell proliferation in the breast leads to breast cancer [3]. It is the most frequently occurring kind of cancer in Ethiopia and the most diagnosed cancer in women [4,5]. Architectural distortion, bulk, calcification, and bilateral asymmetry are the four forms of breast cancer manifestations [4]. Globally, breast cancer ranks as the second most common cause of death for women. Early identification is the most efficient way to lower the death rate from breast cancer. 

### 1.2. Screening Modalities

Breast cancer screening is the first step in the process of breast abnormality identification and breast cancer image analysis, as stated in Debelee et al. [4]. Screening techniques for breast cancer include digital mammography (DM), ultrasound (US), magnetic resonance imaging (MRI), digital breast tomosynthesis (DBT), screen film mammography (SFM), and combinations of the techniques. One of the best and most popular screening techniques for detecting breast tumors now is mammography; yet only a small number of radiologists are qualified to read and diagnose many of the mammograms obtained via population screening [1]. This results in pointless biopsies. As a result, additional methods must be used to investigate and enhance the image, such as identifying the breast border and pectoral muscle detection, which aid in the improvement of images for more detailed breast analysis. Two different methods are used in the analysis of mammograms [2]. The first method consists of a systematic search of every mammogram for visual patterns symptomatic of tumors, and the second is an asymmetric method which involves a systematic comparison of corresponding areas in the left and right breasts. Significant structural asymmetries between the two regions can indicate the possible presence of a tumor. To detect a suspicious mass from digitized mammogram, various pre-processing steps are applied. The pre-processing step will enhance the image’s quality and prepare it for additional processing. Removing the obtrusive regions from the image’s background can further enhance its quality [2]. Because of the dense nature of the breast, detecting the target area can be highly challenging both for the radiologists and the oncologists. Moreover, there is a significant chance of misdiagnosis due to the challenging interpretation of dense breast data. Numerous researchers have made contributions to the CAD research literature to improve radiologists’ mammography diagnosis accuracy and decrease the number of misdiagnoses [6].

ML and DL algorithms are used by CAD systems to accurately detect lesions that are difficult to differentiate with the unaided eye. Nonetheless, several factors may adversely impact these CAD systems’ ability to detect lesions accurately. The pectoral muscle exhibits a pixel intensity that is comparable to the left and right mediolateral oblique muscles, which may lead to misdetection if CAD systems are intended to identify breast cancer [6]. To avoid this, a distinct method for recognizing pectoral muscle is needed. Additionally, one of the main reasons for detecting pectoral muscle in mammograms is that it may be able to identify aberrant axillary lymph nodes, which is a significant indicator of breast cancer. An essential procedure in the CAD system is the removal of the pectoral muscle, as it appears in digital mammography images somewhat brighter than the surrounding breast tissue. The pectoral muscle has roughly the same density as the image’s densely focused tissues. Consequently, to focus the search for breast anomalies in the breast’s “soft tissue” region, the pectoral muscles’ edge processes are crucial [7].

The mammography image is transformed from pixels to binary bits for edge detection. The binary picture’s border points are taken out and overlaid onto the original image. Reduction or sharpening of image attributes, such as borders, edges, or contrast, is referred to as image enhancement. The goal is to increase the processed image’s analytical use. If the enhancement criteria can be precisely articulated, then image enhancement algorithms can be enhanced [4]. Traditional ML and DL algorithms have shown promise in assisting with medical image analysis; however, these methods still face several limitations [3]. ML algorithms often require massive datasets, are prone to errors, and lack interpretability. While DL techniques, particularly CNNs, have advanced the field, they still struggle with efficiently extracting and classifying features from mammogram images due to complex image characteristics and varying levels of tissue density. One critical area in improving mammogram analysis is the accurate segmentation of breast tissue and the removal of the pectoral muscle, which can interfere with the detection of abnormalities.

Current methods for feature extraction and classification, such as the gray-level co-occurrence matrix and Gabor filters, have demonstrated varying degrees of success across different datasets, yet they still fall short in achieving consistently high performance. Given these challenges, there is a need for a more robust and efficient approach to breast cancer detection in mammograms [3,4]. This research proposes that a hybrid CNN model may address these issues. By integrating advanced feature extraction techniques and optimizing classification processes, the proposed hybrid model aims to significantly enhance the accuracy and reliability of breast tissue segmentation and pectoral muscle extraction.

### 1.3. Aims and Objectives of the Proposed Research

The aims and objectives of the proposed hybrid CNN and pruned ensembled extreme learning machine (HCPELM) are as follows:

- Design and develop a CAD model integrating hybrid CNN models to enhance breast cancer detection, segmentation, feature extraction, and classification.
- Implement methods for artifact and pectoral muscle removal to improve the quality and accuracy of mammogram image analysis.
- Utilize the appropriate activation function to enhance feature extraction from mammogram images.
- Aim for state-of-the-art performance in classification and recognition tasks, with a focus on achieving high breast image recognition accuracy.
- Aid healthcare practitioners in the early detection and diagnosis of breast cancer by providing a reliable and efficient CAD system.
- Train and evaluate the designed classifier using the MIAS database and compare its performance against benchmark methods.

By achieving these objectives, the proposed hybrid model aims to provide a reliable and efficient CAD system that can aid healthcare practitioners in accurately diagnosing breast cancer, ultimately contributing to better patient outcomes.

## 2. Related Work

To recognize pectoral muscle in a mammography image, several researchers have devised a variety of image processing approaches [8]. The extraction of pectoral muscle in the medio-lateral oblique (MLO) view of a mammography has been proposed using several approaches [7,8]. The contrast-based method [8] was shown to be less accurate when applied to the full image due to irregular intensity in dissimilar images and light leaking issues in the edges. The authors chose to use several technologies that should offer threshold estimate and automatic contrast computation for that reason [7]. Boundary- and region-based approaches are also among the most used methods for mammography segmentation [3,4,7]. Detection and removal of pectoral muscle are another challenging task in pre-processing. The proposed method identifies the pectoral muscle in MLO mammograms by using Gabor wavelets. This method overcomes the straight-line representation and designs using the Hough transform [7].

The superposition of several muscle types determines the intensity of each pixel in the mammography. The pectoral muscle is among the various breast tissues that are difficult to distinguish because of this superposition [7]. The pectoral muscle is seen as a triangle on one side of the mammography picture. While processing a picture, two important tasks to be completed are image enhancement and de-noising. When it comes to utilizing hierarchical features found in data, deep learning is more beneficial than feature engineering that involves human handcrafting [8]. Shen et al. [9] have used deep learning models to identify, classify, and quantify patterns in medical images. Several algorithms have been communicated recently to perform the de-noising process. Random variables from the Laplace transform are used to display the image using the multi-wavelet with hard threshold approach [9]. There are various frequency levels at which the hard threshold is functional. PSNR (peak signal-to-noise ratio) is used to quantify how well this technique performs. This technique is applied to improve contrast in images and suppress noise. The techniques that have been investigated include adaptive median filters, adaptive mean filters, histogram equalization, histogram-modified local contrast enhancement, extraction of the breast region and pectoral muscles, the contrast limited adaptive histogram equalization (CLAHE) technique, and morphological measures. CAD systems should include several different possibilities for image enhancement, automatic segmentation, and classification. One proposed method for contrast enhancement is the modified histogram method [10]. Using a modified histogram technique, the originally lower-contrast image is improved and normalized. Homomorphic filtering is a technique used to sharpen images. The opacity of pectoral muscle is like that of tumors. Masses and pectoral muscle require almost the same range of intensity due to tiny calcification clusters; this might bias and impact the outcome of any mammography processing method [7]. The pectoral muscle is one of the breast’s high-intensity standards in MLO digital mammography images. Simple histogram thresholding is one of the first methods to challenge the separation of the breast region used a simple threshold to part the breast from the background. Other methods use a combination of local thresholding, region growing, and morphological filtering [7,8].

The pectoral muscle is one of the high-intensity landmarks in breast digital mammography in MLO digital mammography images [7]. The upper right or left corner of the chest’s triceps is known as the pectoral muscle region. Its opacity is comparable to that of tumors [3]. Masses and pectoral muscle have nearly identical intensities due to macrocalcification clusters; this can skew and impact the results of any mammography processing technique [9]. Because of this, to obtain a suitable and precise area of interest (ROI), it is frequently required to automatically detect and remove the pectoral muscle. The primary marker for matching mammograms taken from various angles is the pectoral muscle. The pectoral muscle varies in size, intensity, shape, and texture due to the architecture and patient placement during picture capture [10]. Furthermore, the pectoral muscle edge may be hidden by the fibroglandular tissue. For all these reasons, automated pectoral muscle segmentation is a difficult and demanding task. Among the notable anatomical characteristics of the pectoral muscle are its roughly triangular shape, noticeable variation in intensity between the pectoral region and breast tissue, and gentle tapers from top to bottom. This work considers an enhancement filter and frontier evolution strategy to automatically determine the pectoral muscle boundaries [4]. DL is also helpful in abnormality detection, cancer/tumor segmentation, and classification in addition to recognizing the objects from images [8]. Suzuki [6] has used DL in medical imaging analysis to evaluate the changes that occurred prior to and following the implementation of deep learning. The author has also disclosed that, in contrast to using machine learning methods for image analysis, deep learning is effective in medical image analysis. 

### 2.1. Segmentation and Classification Performance Metrics

According to the research literature, commonly used metrics that are generally successful in describing classification performance are area under curve (AUC), specificity (Sp), sensitivity (Sn), positive predictive values (PPV), accuracy (Acc), recall (R), precision (P), geometric mean (G-Mean), and Matthew’s correlation coefficient (MCC) [10,11]. The best criteria for assessing the segmentation performance [7] of the algorithms are performance measures such as Hausdorff distance (H) and Zijdenbos similarity index (ZSI) or F1-score, intersection over union (IoU), and Dice similarity coefficient (DSC). The accurately classified pixels within the correctly labeled classes in classification models represent the true positives for the segmentation process.

### 2.2. Datasets

Based on the various imaging modalities, many datasets are ready for medical image analysis. The most widely used and accessible datasets for breast cancer are those related to mammography and histology. Table 1 discusses some of the most popular datasets.

### 2.3. Deep Learning for Detection of Breast Cancer

A deep-learning-based system for detecting breast cancer was proposed by Shen et al. [2] utilizing an end-to-end training strategy with mammograms from the INbreast and Digital Database for Screening Mammography (DDSM) databases. ResNet-50 and VGGNet-16 were the deep learning architectures employed in their paper. At the single-model and four-model (ResNet-ResNet, Res-Net-VGGNet, VGGNet-VGGNet, and VGGNet-ResNet) averaging levels, the suggested strategy was assessed in terms of AUC. The best single model for the DDSM dataset had a per-image AUC of 0.88; four-model averaging increased the AUC to 0.91 with 86.1% sensitivity and 80.1% specificity. The optimal single model for the INbreast database has a per-image AUC of 0.95; however, four-model averaging produced an improved AUC value of 0.98 with 86.7% sensitivity and 96.1% specificity.

Wu et al. [18] suggested a DCNN architecture to identify mammography-based breast cancer screening tests based on four columns of ResNet-22. Over one million photos were used in all 200,000 exams to test and train the suggested DCNN model. When evaluated on the screening population, the network’s performance was able to predict whether breast cancer exists with an AUC of 0.895. The results were compared to the readings of 14 radiologists. Alzubaidi et al.’s [19] suggested 74-layer CNN uses a transfer learning technique. The erythrocytesIDB dataset, which contains pictures of peripheral blood smear samples from sickle cell disease patients, served as the model’s pre-training dataset. The original microscope image was separated into 12 patches, and the most often occurring patch label was selected as the image label through majority voting. At the patch level, the model’s accuracy was 90.5%, and at the image level, it was 97.4%. They misused the majority voting process since, although most cells are normal, the algorithm may still designate any diseased cells as normal, which is undesirable. Two deep learning techniques were presented by Zhu et al. [20] to forecast the presence of invasive carcinoma on MRI scans. The first method used a pre-trained GoogleNet model with transfer learning to predict the presence of aggressive cancer. In a second method, the authors employed SVM to predict the invasive disease by extracting features from the natural photos. With the transfer learning strategy, the best classification result was obtained in terms of AUC, which was 0.53, and with extracted features, it was 0.70. Li et al. [21] investigated the mass classification capabilities of full-field digital mammography (FFDM) and digital breast tomosynthesis (DBT) using deep neural networks with or without transfer learning. Additionally, they investigated a viable DBT and FFDM combination technique to improve classification performance. For the 2D photos, they used an 11-layer deep convolutional neural network (DCNN) and a 16-layer VGG network (VGG-16). To handle the additional dimension in the 3D DBT images, they modified the 11-layer DCNN. The most effective of these techniques was a 2D-DCNN that was trained by fusing the DBT and FFDM. It performed best on three class classifications (benign, malignant, and normal), with average AUC, accuracy, sensitivity, and specificity of 0.95, 92.13%, 83%, and 93.84%, respectively. Zeiser et al. [22] investigated the use of the U-Net model for mass segmentation on mammograms at various depths, either with or without data augmentation. On the DDSM dataset, the U-Net model with a depth of 5 and data augmentation proved to be the most effective, exhibiting sensitivity of 92.32%, specificity of 80.47%, accuracy of 85.95%, Dice index of 79.39%, and AUC of 86.40%. To classify breast cancer, Shen et al. [9] used a group of four of the strongest deep learning models, which were built using Resnet50 and VGG16 as patch classifiers and Resnet and VGG blocks as the top layer. The combination of these classifiers produced the best AUC of 0.91 (sensitivity: 86.1%, specificity: 80.1%) when it came to the DDSM dataset’s classifications and detection of benign and malignant tumors. For segmenting and extracting adipose tissue, fibroglandular tissue (FGT) inside the breast, and all non-breast tissues outside the breast from breast MRIs, Zhang et al. [23] employed U-Net architecture. For breast and FGT, they obtained mean DSC of 0.95 and 0.91, respectively, and mean accuracy of 0.98 and 0.97 for each. Zhou and colleagues [24] employed 3D deep CNN, whereas Huang and colleagues [25] employed 3D DenseNet architecture consisting of 37 layers to diagnose breast cancer and locate lesions in dynamic-contrast-enhanced (DCE) magnetic resonance imaging (MRI) data through a weakly supervised approach. The algorithm performed as follows [25]: 83.7% accuracy, 90.8% sensitivity, 69.3% specificity, 0.859 AUC, and 0.501 Dice distance were recorded for the detection of breast cancer.

The extreme learning machine (ELM) is a feed-forward neural network that may be used for feature learning, classification, regression, clustering, compression, and other tasks without requiring the hidden node parameters to be changed. It can have a single layer or several layers of hidden nodes. These hidden nodes may never be changed, be assigned at random, or inherit from their predecessors [26]. A fast-learning method is essentially produced when the weights of hidden nodes are often learned in a single step. Compared to backpropagation networks, ELMs can learn thousands of times faster and achieve good generalization performance. These models perform better in classification and regression applications than support vector machines. ELM is used to handle a variety of health disease detection and prediction problems [27]. The ELM algorithm determines the input weights and biases at random. Bacterial colony optimization (BCO) is used to optimize the weights and bias. Three separate metrics are used to calculate the performance of the BCO+ELM, which is applied to heart disease prediction, and from the experimental results, it is observed that the BCO+ELM achieved better results [27].

A single ELM classifier or a set of ELM binary classifiers have been proposed by Rong et al. [28]. The multi-class problem is divided into a two-class problem when binary ELM classifiers are used. This is done by applying the one-against-all (OAA) and one-against-one (OAO) schemes, which are abbreviated as ELM-OAA and ELM-OAO, respectively. The multi-class problem is implemented in a single ELM classifier using an architecture of multi-output nodes equal to the number of pattern classes. A few multi-class benchmark problems are used to assess their performance, and the simulation results demonstrate that ELM-OAA and ELM-OAO require fewer hidden nodes than the single ELM classifier. Additionally, when the pattern class labels are not greater than,10 ELM-OAO typically has a computation overhead that is comparable to or less than 10 times that of the single ELM classifier. An automated and systematic approach to building ELM networks is the pruned extreme learning machine (P-ELM), as proposed by Rong et al. [29]. This is because employing an excessive number of hidden nodes could generate issues with underfitting or overfitting in pattern classification. P-ELM began with many hidden nodes, and as it learned, it eliminated those that were unnecessary or only marginally significant by considering how relevant they were to the class labels. As a result, ELM’s architectural design can be automated. Simulation findings showed that the P-ELM produced compact network classifiers with robust prediction accuracy and fast reaction on unseen data, as compared to the traditional ELM.

### 2.4. Breast Histopathology Image Analysis

The confirmation of malignant sales identified by other imaging modalities is aided by breast histology. Identification of the malignant cells on histology slides is a laborious and time-consuming task because the slides may include millions of cells. By automating the diagnostic pathology workflow, machine learning and computer vision techniques can cut down on analysis time [30]. A multi-scale input and multi-feature network (MSI-MFNet) model combines multi-resolution hierarchical feature maps from the network’s dense connectivity structure to learn the general structures and texture features of various scale tissues. Using two publicly available benchmark datasets, the MSI-MFNet predicts the likelihood of an illness on the patch and shows improved accuracy, specificity, and sensitivity. Using only image information, an automatic image normalization method [31] corrects the intensity of pictures from breast dynamic-contrast-enhanced magnetic resonance imaging (DCE-MRI) obtained by several MRI scanners with varying imaging parameters. This study used DCE-MRI pictures from 460 breast cancer patients that were obtained using several scanners. Three T1-weighted post-contrast images and one T1-weighted pre-contrast image were available for each subject. The normalization technique worked on the premise that a single voxel value should reflect the same type of tissue across various patients. Normalization is anchored by four different tissue/material types: (1) air; (2) fat tissue; (3) dense tissue; and (4) heart. Accurate tissue segmentation was achieved using a DL-based method, and to normalize the same type of tissue in various patients into the same intensity ranges, a subject-specific piecewise linear mapping function was applied between the anchor points. The approach yielded much increased consistency in pixel values and extracted radiomics characteristics, with 300 subjects used for training and the remaining subjects utilized for testing [31].

By eliminating the pooling layers from the fourth Dense-block and passing the derived feature maps from each Dense-block to the squeeze-and-excitation (SENet) module for breast histopathology pictures, Li et al. [32] altered the Densenet-121 design. To obtain additional channel-specific information, they employed SENet. They employed a fully connected layer for categorization after concatenating each SENet output. They employed the transfer-learning method to apply a pre-trained Densenet model for their architecture. Their system achieved an average accuracy of 88% for binary classification across various magnification settings using the publicly available BreakHis dataset [32]. For the classification of breast cancer histopathological images, a hybrid convolutional and recurrent deep neural network [33] was used. This approach combined the benefits of convolutional and recurrent neural networks (RNN) based on multilevel feature representation of the histopathological image patches, preserving both the short- and long-term spatial correlations between the patches. According to the experimental data, it completed the four-class classification task with an average accuracy of 91.3%. A dataset of 3771 histological pictures of breast cancer has been made available to the scientific community by both us and them. It can be accessed by the public at http://ear.ict.ac.cn/?page_id=1616 accessed on 14 March 2024. With as many distinct subclasses across a wide range of age groups as feasible, this dataset offers significant variation in data to address the issue of benign image classification accuracy being rather low. Pre-trained deep learning networks were investigated by Sharma et al. [34] as a feature extractor from images of breast cancer histology. To leverage the pre-existing networks (VGG16, VGG19, and ResNet50) as feature extractors, they employed transfer learning on them. An SVM classifier was then used to classify the retrieved features. The most accurate VGG16 network was one which used linear SVM (93.79% for magnifications of 40×, 92.92% for 100×, 91.23% for 200×, and 91.79% for 400×). In their proposal, Vang et al. [35] used inception-V3 to support a deep learning technique with reinforcement backing for multi-class (normal, benign, in situ, and invasive) classification. Logistic regression, gradient boosting machine (GBM), and majority voting were used in the ensemble fusion strategy for image level prediction. Their method performed poorly for both the benign and normal classes in terms of sensitivity. The addition of a dual path network (DPN) to be used as a feature extractor increased the sensitivity of the benign and normal predicted classifications. To further improve predictions, the retrieved features are transferred to the next layer of ensemble prediction fusion, which uses SVM, logistic regression, and GBM. This method received an accuracy score of 87.5%. Two techniques for zero watermarking algorithms for medical image authentication have been developed by the authors [36]. Singular-value decomposition (SVD) and composite CT–SVD domain are the methods used to secure the images. Medical photos are used in the experiments, and this model is appropriate for healthcare facilities since it has a robust method that can withstand several attacks and makes it easier to authenticate the photographs. They also presented guidelines for sharing clinical results with the radiologists who are remotely located, and their methods are more robust than other zero watermarking schemes.

Jaganathan Dhayanithi et al. [37] introduced a transfer-learning-based classifier to classify breast cancer histopathology. Examples of transfer-learning-based concatenated pre-trained models intended for classification applications include the VGG-16, ResNet50, MobileNetV2, and DenseNet121 convolutional neural network architectures. Using histopathology data, the concatenated classification model was tested against separate classifiers and was able to attain a 98% training accuracy. Our studies’ results demonstrate how effective our four-level concatenated model is in improving the precision of data analysis related to breast cancer histopathology. By improving pathologists’ diagnostic skills, this methodology helped patients with breast cancer receive more personalized and informed treatment planning [37]. The rectified linear unit (ReLU) activation function was changed in the modified LeNet design [38], along with batch normalization, to stabilize the training process and improve performance in smaller, less complex CNN architectures like LeNet. Internal covariate shift, a phenomenon where the distribution of activations inside a network changes during training, is addressed by batch normalization. These adjustments aid in reducing computation time and preventing overfitting. The model’s performance is assessed using an extensive dataset, and it is compared to other pertinent deep-learning models. With a 96% accuracy rate in pneumonia picture recognition, the results show a high recognition rate, and the model may help with better treatment choices and patient outcomes [38]. Better recognition accuracy is achieved by solving the multiview object recognition issue using a wrap CNN (Wrap-CNN) [39] with a voting method. Three stages make up this model: voting schemes, pre-processing, and pre-training CNNs. The pictures are categorized into the appropriate classes using these pre-trained CNN models as feature extractors. Nine pre-trained CNN models are employed in tandem in Wrap-CNN: Alex Net, VGG-Net, GoogLeNet, Inceptionv3, SqueezeNet, ResNet v2, Xception, MobileNetV2, and ShuffleNet. Ultimately, a voting system is used to select the output class from the nine anticipated classes. The system’s accuracy was tested in two different scenarios, including images with and without rotation. Its results show that it can recognize objects in multiview using an industrial automation system with 99% and 93% accuracy, respectively [39]. A privacy-enhanced generative adversarial network (PrEGAN) for EMR data training and realistic mapping model generated and discriminated the ground truth with generated mask via a computation of loss function for de-identification or removal of personal linked/connected data [40]. The objective of this model is to generate the mask of EMR, which is realistic and like the ground truth. The model is trained and validated with two distinguished discriminators, the CNN-based discriminator and neural networks’ textural data generator for medical images. The experimental results demonstrated a higher degree of data privacy and de-identification in EMR with 88.32% accuracy [40].

The authors [41] have proposed the adaptive teaching–learning-based (ATLB) heuristic to identify optimal hyperparameters for various network architectures. The study focused on three types of deep neural networks for classification: RNN, long short-term memory (LSTM), and bidirectional long short-term memory (BiLSTM). The evaluation of the proposed ATLB method is conducted using different learning rate schedulers: cyclical learning rate (CLR), hyperbolic tangent decay (HTD), and a technique that toggles between hyperbolic tangent decay and triangular mode with restarts (T-HTR). Experimental results have demonstrated performance improvements on the 20Newsgroup, Reuters Newswire, and IMDB datasets.

The authors have designed a deep-learning-based classification model aimed at reducing misclassifications and handling large datasets [42]. Images are filtered using the adaptive guided bilateral filter, and texture and edge attributes are extracted with the spectral Gabor wavelet transform. The black widow optimization method selects the best features, which are then fed into a red-deer-optimization-enhanced gated deep reinforcement learning network model for classification. The model was tested on the brain tumor MRI dataset using the MATLAB platform, achieving an accuracy of 98.8%.

Barbieri et al. [43] have reported the case of a female patient, age 52, who had a parietal skin lesion removed. Analyses using immunohistochemistry and histology pointed to the genesis of breast cancer. The primary tumor was not found despite a complete clinical assessment, as well as laboratory and radiographic testing. The patient was offered hormone medication, but she turned it down. After 28 months, the patient complained of a lump in her right cervical region. A full-body positron emission tomography (PET) scan demonstrated the spread of the illness to the bone and lymph nodes. As a result, metastases from an unknown primary location to several organs are indicative of a diagnosis of carcinoma of unknown primary (CUP) syndrome.

A particular type of CUP known as occult breast cancer (OBC) is described as clinically identifiable metastatic carcinoma that results from an undetected initial breast tumor in a case report by the authors [43]. OBC makes up 0.3–1% of all cases of breast cancer and frequently shows signs of skin, bone, and lymph node metastases.

The first steps in the diagnosis of CUP syndrome from OBC are radiological and clinical exams. Nevertheless, histological and immunohistochemical studies, assessment by a multidisciplinary team, and a thorough therapeutic approach are necessary for a conclusive diagnosis and course of treatment [43]. OBC’s CUP syndrome was verified. A multimodal treatment strategy was started, involving biological therapy, hormone therapy, and radiation. The patient is still asymptomatic five years after the original presentation, even though the disease has spread.

### 2.5. Relevance of CAD and HCPELM

#### 2.5.1. Enhanced Detection and Diagnosis

Traditional clinical and radiological methods failed to identify the primary breast tumor. The HCPELM model, designed for superior accuracy in detecting and classifying breast cancer, could enhance the detection capabilities. By leveraging convolutional layers for feature extraction and fully connected layers for classification, HCPELM can potentially identify subtle patterns indicative of OBC that might be missed by conventional methods.

#### 2.5.2. Improved Feature Extraction

Accurate detection of tumor, metastases, and their primary origin is critical, and is challenging with conventional imaging alone. HCPELM’s advanced feature extraction capabilities, particularly its convolutional layers, can analyze medical images to detect minute anomalies and complex features. This could assist in locating the primary tumor or in providing a more accurate assessment of metastases, even when the primary site is not visible.

#### 2.5.3. Predictive and Personalized Medicine

Tailoring treatment to individual patients is essential, especially when standard therapies are declined, as in the case report. By analyzing large datasets, HCPELM can identify patterns and predict outcomes based on individual patient data. This can help in developing personalized treatment plans, potentially offering alternatives when standard options are refused.

#### 2.5.4. Continuous Monitoring and Follow-Up

Continuous monitoring of disease progression and treatment response is crucial, as evidenced by the patient’s disease dissemination 28 months later [43]. CAD systems using HCPELM can facilitate regular monitoring through follow-up imaging analysis, helping to track disease progression and adjust treatment plans accordingly.

#### 2.5.5. Practical Applications of the Proposed Model

- HCPELM initial diagnosis: HCPELM could have been utilized during the initial presentation to analyze skin lesion images and other scans more effectively, possibly identifying subtle signs of OBC.
- Monitoring disease progression: Following the refusal of hormone therapy, HCPELM could assist in regular imaging follow-ups, providing early detection of disease spread.

Integrating a CAD model like HCPELM in disease management could significantly enhance diagnostic accuracy, personalize treatment, and monitor disease progression. The advanced capabilities of HCPELM in feature extraction and data integration make it a valuable tool in addressing complex challenges and ultimately improving patient outcomes.

## 3. Proposed Breast Segmentation and Pectoral Muscle Extraction Using Hybrid CNN and Pruned Ensembled Extreme Learning Machine (HCPELM)

**Figure 1:**
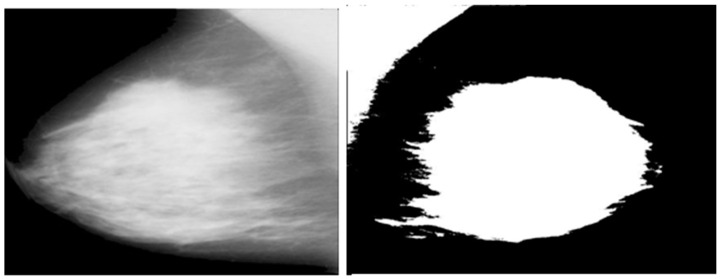
Raw mammogram image and pectoral muscle removed image.

The DL model is implemented in this paper for the segmentation of breast and pectoral muscle removal. The mammogram image is presented in Figure 1. To identify the pectoral muscle, image pre-processing is essential to eliminate noise and artifacts from a mammogram image. This proposed methodology consists of three stages: (i) image de-noising, (ii) breast segmentation, and (iii) pectoral muscle extraction. The raw mammogram image is usually contaminated with numerous noise sources. Elimination of noises from a mammogram image increases the overall system performance. The second and third stages represent breast segmentation and pectoral muscle extraction. The pectoral muscle removal process is as follows; grayscale conversion simplifies the image to one channel, making processing easier.

Gray(x,y) = 0.299·Red(x,y) + 0.587·Green(x,y) + 0.114·Blue(x,y)(1)

In Equation (1), the conversion of a color image to a grayscale image is performed by taking a weighted sum of the red, green, and blue channels: the weights 0.299, 0.587, and 0.1140 are used to reflect the perceived luminance of each color channel to the human eye. These weights are derived from the luminance model of human vision and represent how sensitive the human eye is to each of the primary colors. Red (0.299): The red channel is less sensitive to the human eye compared to green but more sensitive compared to blue. Therefore, it has a moderate weight. Green (0.587): The green channel is the most sensitive to the human eye, which is why it has the highest weight. The human eye perceives green light more intensely than red and blue light. Blue (0.114): The blue channel is the least sensitive to the human eye, hence it has the lowest weight. The human eye is less responsive to blue light compared to red and green. These weights ensure that the grayscale image accurately represents the perceived brightness and details of the original color image, considering human visual perception.

Thresholding helps in distinguishing the foreground (pectoral muscle) from the background.

Binary(x,y) = 255 if Gray(x,y) > T, otherwise Binary(x,y) = 0 if Gray(x,y) ≤ T (2)

T is a threshold value (e.g., 200). This converts the grayscale image to a binary image where pixels above the threshold are set to 255 (white) and others to 0 (black). Contour detection identifies the edges of objects in the image.

Contours are detected using algorithms such as the border-following algorithm (e.g., Suzuki and Abe’s method). This step involves tracing the boundaries of connected components (i.e., regions of white pixels) in the binary image. Area A of each contour is calculated. The contour with the largest area is selected, assuming it corresponds to the pectoral muscle.

(3) $$A=\sum_{(x,y)\in contour}1$$

Masking and removal techniques are used to effectively eliminate unwanted parts of the image. A mask is created by drawing the selected contour filled with white (255) on a black (0) background. The mask is applied to the original image to isolate and remove the pectoral muscle.

Creating a mask: A mask is created by drawing the selected contour filled with white (255) on a black (0) background. The mask is applied to the original image to isolate and remove the pectoral muscle.

Applying the mask:

(4) $$Result\left(x,y\right)\left\{\begin{array}{c} \mathrm{Image}\left(x,y\right)\mathrm{if}\;\mathrm{Mask}\left(x,y\right)=0\\ 0\;\mathrm{if}\;\mathrm{Mask}\left(x,y\right)=255 \end{array}\right\}$$

**Figure 2:**
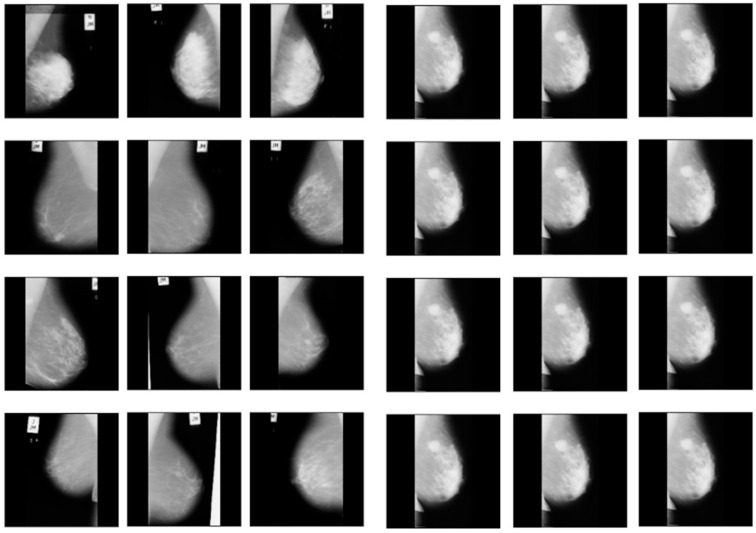
Before and after removal of pectoral muscles.

This operation keeps the pixel values of the original image where the mask is black and sets them to zero where the mask is white. The process is automated to handle multiple images and save the results efficiently. The following Figure 2 shows the significance of pectoral muscle removal.

The proposed model is divided into two parts, the first of which is to implement hybrid CNN and apply the pectoral-muscle-removed breast dataset. The model is designed as a hybrid CNN due to the following reasons:

- Convolutional and fully connected layer combination: The model combines fully connected layers (Dense) for classification and convolutional layers (Conv2D) for feature extraction. This is a standard architecture for CNNs but calling it “hybrid” emphasizes the integration of these different types of layers.
- Use of batch normalization: Batch normalization layers are included after each convolutional and fully connected layer. This technique helps in normalizing the activations and gradients, which speeds up training and provides some regularization effect. The inclusion of batch normalization is an enhancement over traditional CNN architectures.
- Dropout for regularization: The model incorporates dropout layers to prevent overfitting. Dropout is a regularization technique that helps the model generalize better by randomly setting a fraction of input units to zero during training.
- Data augmentation: The use of a data generator (datagen.flow) for augmenting the training data introduces additional variability in the training set, which can help the model generalize better. This is not part of the model architecture itself, but it contributes to the hybrid nature of the training process.

The hybrid elements are convolutional and fully connected layers. The convolutional layers extract spatial features from the input images. These layers capture patterns like edges, textures, and more complex features in deeper layers. The fully connected layers take these features and combine them in a non-linear manner to perform the final classification. Batch normalization normalizes the inputs of each layer to have zero mean and unit variance. This stabilizes and speeds up the training process by mitigating the problem of internal covariate shift. Dropout randomly drops units (along with their connections) during training to prevent the network from becoming too reliant on specific neurons. This forces the network to learn more robust features.

Data augmentation artificially increases the size of the training set by creating modified versions of the images. Techniques such as rotation, flipping, scaling, and shifting are used. This enhances the model’s capacity to generalize to fresh, untested data. The advantage of hybrid CNN is enhanced performance in which the model integrates multiple techniques like batch normalization, dropout, convolutional, and fully connected layers. By using data augmentation and dropout, the model improves its ability to generalize, which is a characteristic of hybrid models aiming to leverage various methods for better performance. Batch normalization helps in stabilizing and speeding up training, making the model more robust.

The entire process of hybrid CNN is as follows:

- Importing the required libraries: Batch normalization is imported from tensorflow.keras.layers, which will be used to normalize the activations of the previous layer and to improve training.
- Creating the hybrid CNN model: The model is created as an instance of Sequential, which means layers are added one after the other; in the hybrid CNN model, convolutional layers are used to extract features from input images. The layers in the model:
  ◦ First convolutional block:
    ▪ Conv2D(32, kernel_size = (3, 3),activation = ‘relu’,input_shape = (64, 64, 1));
    ▪ Adds a 2D convolutional layer with 32 filters of size 3 × 3, using ReLU activation;
    ▪ input_shape = (64, 64, 1) specifies the shape of the input images (64 × 64 pixels, 1 channel for grayscale);
    ▪ BatchNormalization()
    ▪ Normalizes the activations of the previous layer to improve training stability and performance;
    ▪ MaxPooling2D(pool_size = (2, 2));
- Max pooling reduces spatial dimensions to retain important features while reducing computational load. A max-pooling layer with a 2 × 2 pool size is added to reduce the spatial dimensions of the feature maps.
  ◦ Second convolutional block:
    ▪ Conv2D(64, kernel_size = (3, 3), activation = ‘relu’);
    Adds another convolutional layer with 64 filters of size 3 × 3, using ReLU activation.
    BatchNormalization()
    MaxPooling2D(pool_size = (2, 2))
  ◦ Third convolutional block:
    ▪ Conv2D(128, kernel_size = (3, 3), activation = ‘relu’):
    ▪ Adds a convolutional layer with 128 filters of size 3 × 3, using ReLU activation.
    ▪ BatchNormalization()
    ▪ MaxPooling2D(pool_size = (2, 2))
    ▪ Fully connected layers combine extracted features to make predictions.
  ◦ Flattens the 3D feature maps into a 1D vector to prepare for the fully connected layers.
    ▪ Dense(128, activation = ‘relu’):
    ▪ Adds a fully connected (dense) layer with 128 neurons, using ReLU activation.
  ◦ BatchNormalization()
    ▪ Dropout(0.5): Dropout is used to prevent overfitting by randomly deactivating neurons during training.
    ▪ Adds a dropout layer to prevent overfitting by randomly setting 50% of the neurons to zero during training.
    ▪ Dense(64, activation = ‘relu’):
    ▪ Adds another dense layer with 64 neurons, using ReLU activation.
    ▪ BatchNormalization()
    ▪ Dense(2, activation = ‘softmax’):
    ▪ Adds the output layer with two neurons (assuming a binary classification problem), using softmax activation to output probabilities for each class;
- Compilation: The compilation step involves configuring the model with an optimizer, loss function, and metrics, followed by training the model with data augmentation and evaluating it on validation data. The model is compiled using the following command: cnn_model.compile(optimizer = Adam(), loss = ‘sparse_categorical_crossentropy’, metrics = [‘accuracy’]). This compiles the model with the Adam optimizer, uses sparse categorical cross-entropy loss, suitable for integer-labeled classes, and tracks accuracy as a metric during training.
- Training the model: cnn_model.fit(datagen.flow(xtrain, y_train, batch_size = 32), epochs = 50, validation_data = (xtest, y_test)). This trains the model using data augmentation (datagen.flow). xtrain and y_train are the training data and labels. batch_size = 32 specifies the batch size for training. Epochs = 50 sets the number of training epochs. Validation data = (xtest, y_test) provides the validation data and labels for evaluating the model during training.

The extreme learning machine (ELM) is a single hidden-layer feed-forward neural network (SLFN). To execute higher learning, the SLFN’s performance needs to be acceptable for modeling the system for inputs like threshold value, weight, and activation function [28]. Gradient-based learning approaches include iteratively changing each of these parameters until they reach the desired value. As a result, the performance may yield poor results since it may become tied to the slow and local minimum. Whereas the input weights are chosen at random, the output weights are determined analytically in the ELM learning process, in contrast to FNN, which is refreshed based on the gradient. The success rate of an analytic learning process rises because the likelihood of being fitted to a local minimum can be significantly decreased by the resolution time and the error magnitude. ELM can be utilized to choose a linear function or non-linear (sigmoid and sinusoidal), non-derivatized, or intermittent activation functions for the cells in the hidden layer. 

The pruned extreme learning machine (PELM) algorithm is an advanced variant of the traditional ELM designed to enhance performance by selectively removing less significant neurons from the model. The procedure begins by initializing an ELM with a specified number of hidden neurons and randomly assigning weights between the input and hidden layers. The ReLU activation function is applied to transform the input data. During the training phase, the hidden layer’s output matrix is computed, and its pseudoinverse is used to determine the weights between the hidden and output layers. This completes the initial training of the ELM.

Following this, a pruning step is introduced to refine the model. The pruning process involves calculating the maximum absolute weights of the connections between hidden neurons and the output layer. Neurons with weights below a predefined threshold (e.g., 0.05) are considered insignificant and are pruned. This threshold can be adjusted based on the desired level of pruning, with typical values being 0.01, 0.02…, 0.05. The pruned model retains only the significant neurons, leading to a more efficient and potentially more accurate model. The threshold of 0.05 can be set as appropriate based on empirical evaluation, where different thresholds are tested, and the resulting model performances are compared on validation data. This threshold balances retaining significant neurons for accurate predictions and removing those with minimal impact, optimizing both model efficiency and performance.

To further improve generalization and robustness, an ensemble of PELMs can be used. Multiple ELM models with varying numbers of hidden neurons are trained and pruned independently. Predictions from these individual ELMs are averaged to produce the final prediction, leveraging the strengths of each model. This ensemble approach helps to mitigate the weaknesses of individual models and enhances overall performance. The effectiveness of the pruned ELM and its ensemble is evaluated using metrics like accuracy, demonstrating the approach’s ability to produce high-quality predictions with reduced computational complexity. The PELM Algorithm 1 is as follows:
**Algorithm 1** Pruned ELM ProcedurePruning threshold 0.05 (0.01, 0.02, 0.05)Assign max weight higher thresholdIdentifying significant neuronsImport matplotlib and seaborn librariesDefine class PrunedELMCalculate the maximum absolute weight for each hidden neuronIdentify neurons with weights below the thresholdPrune the insignificant neuronsOne-hot encode the labels for ELM trainingInitialize and train the ELMPrune the ELMCombine features selection and training through pruned ELMRunning ensemble with multiple ELMCalculate average predictions

## 4. Experimental Results

The present study exploits medical images that are gathered from the Mammographic Image Analysis Society (MIAS), which is the benchmark database for mammogram analytics. The database holds 322 mammogram images, each 1024 × 1024 pixels in size, and 8-bit gray-level images [13]. The 322 mammogram images are classified by radiologists using the Breast Imaging Reporting and Data System. The raw MIAS images have a spatial resolution of 50 µm per pixel.

When conducting studies, researchers frequently use publicly available datasets or publish their own datasets. For instance, a dataset containing 3771 histological pictures of breast cancer was made available by the authors and is currently accessible to the public at [33]. This dataset provides significant data diversity to address the problem of relatively low classification accuracy for benign photos, as it covers a variety of subclasses across different age ranges. We are using benchmark datasets that are currently accessible in this study [13].

For the classification of breast cancer histopathological images, a hybrid convolutional and recurrent deep neural network [33] was used. The short- and long-term spatial correlations between patches were preserved, and this method merged the benefits of convolutional and RNN based on multilayer feature representation of the histopathological image patches.

According to the experimental findings, it completed the four-class classification test with an average accuracy of 91.3%.

### 4.1. Artifact Removal and Breast Region Segmentation

**Figure 3:**
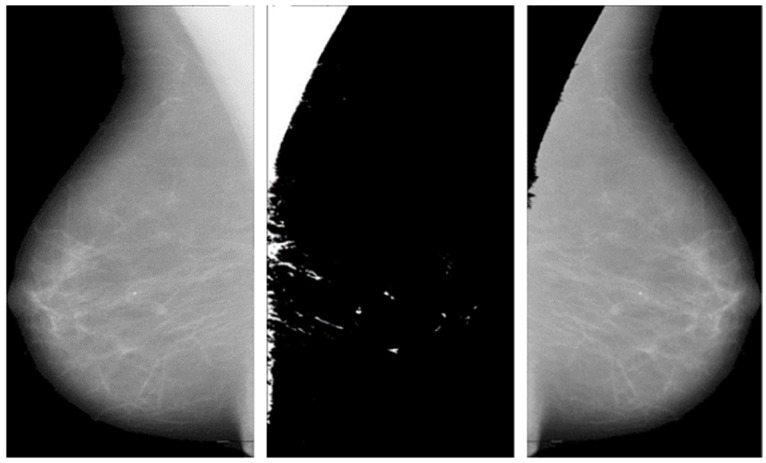
Pectoral muscle removal.

**Figure 4:**
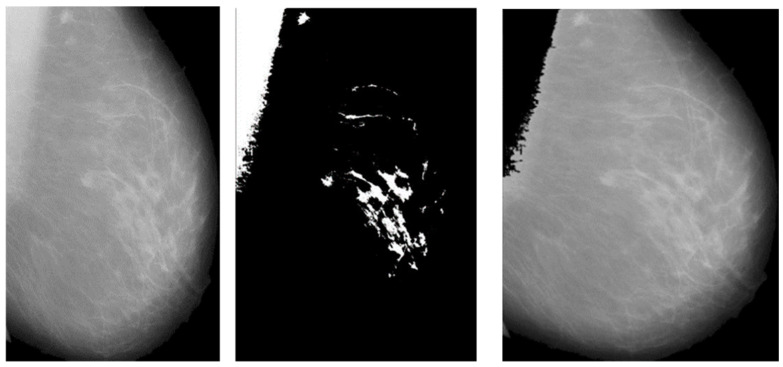
Results of pectoral muscle removal.

The artifacts cover an average of 70% of the area of the mammogram, and the remaining 30% area only covers the breast region. In this stage, artifacts are removed with threshold and morphological operations. The initial step of region segmentation is conversion of grayscale image to a binary image. Binarization is accomplished with the Otsu threshold technique. From the connected component labeling, the largest pixel (component) is selected, and the remaining components are removed to produce higher accuracy and reduced execution time, the breast region is extracted, and further analysis is performed with this extracted region. Figure 3 and Figure 4 show the artifact removal and breast region segmentation.

The final stage of pre-processing is elimination of pectoral muscles from the mammogram image. Contingent upon the image orientation, pectoral muscle will be positioned in the upper left or upper right corner. Tissue density and the dense area of pectoral muscle are tightly associated, and this relationship may influence subsequent mammography analysis. Pectoral muscle detection and removal are crucial steps in the pre-processing of mammograms.

**Figure 5:**
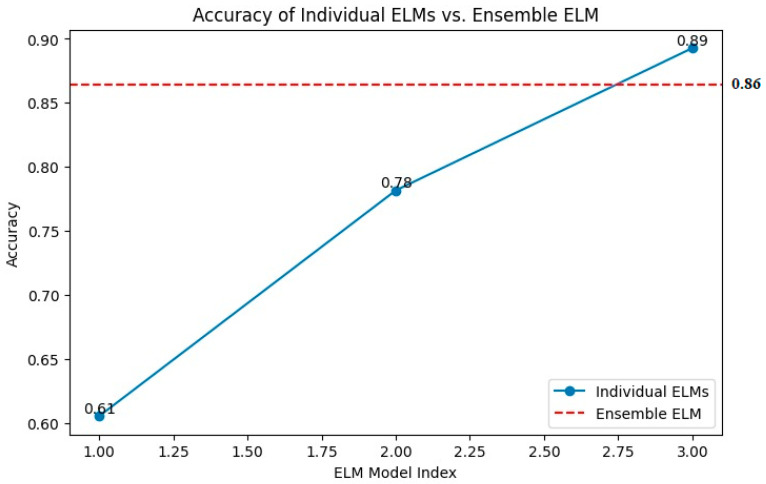
Accuracy of individual versus ensemble ELM.

The comparison highlights the performance advantage of the ensembled ELM over individual ELMs. While certain of the (individual) ELMs demonstrate relatively high accuracy, such as the one with 89%, the ensembled ELM offers a more consistent and robust performance with an accuracy of 86%, as presented in Figure 5. This suggests that combining multiple learning machines in a hybrid and cooperative manner can produce more reliable and accurate results than relying on a single model. The consistency and robustness of the ensembled ELM is crucial for practical applications where reliable predictions are essential. The superior performance of the ensembled ELM in terms of accuracy is particularly valuable in medical imaging, such as predicting target areas of cancer in mammogram images.

Higher accuracy in predictions directly translates to better detection of cancerous regions. This reduces the likelihood of false positives and false negatives, ensuring that more cases of cancer are correctly identified, and fewer healthy regions are mistakenly flagged as cancerous. The ensembled ELM’s robust performance offers more consistent results, which is critical in medical diagnostics. Consistency ensures that different images of the same patient yield similar predictions, aiding in reliable monitoring and assessment. With a high-performing model like ensembled ELM, radiologists and oncologists can have greater confidence in the automated analysis provided by the system. This can assist in making informed decisions regarding further testing, biopsies, or treatments. Automated systems with high accuracy can significantly reduce the time required to analyze images compared to manual examination by medical professionals. This efficiency can be particularly beneficial in settings with high patient volumes. The ensembled ELM’s superior accuracy and reliability make it a powerful tool in the early detection and precise targeting of cancerous areas in mammogram images, ultimately contributing to better patient outcomes.

**Figure 6:**
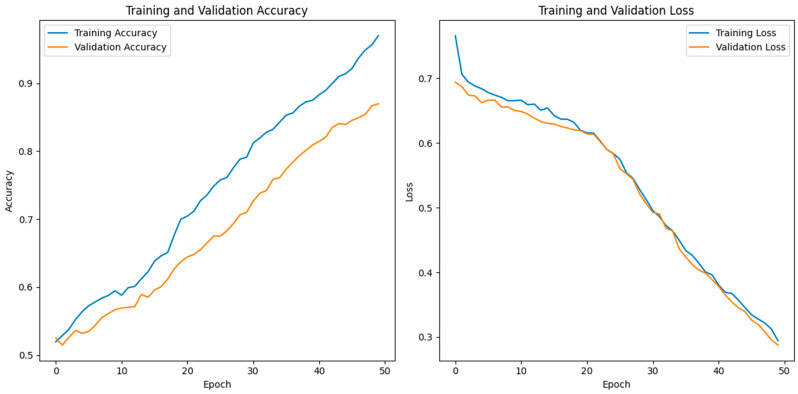
Training versus validation accuracy of the proposed HCPELM model.

The reported training and validation metrics for the hybrid CNN indicate a model that performs well during training but faces some challenges with generalization. A training accuracy of 96.97% and a low training loss of 0.2944 suggest that the model has learned the training data quite effectively, as presented in Figure 6.

The model’s architecture, which combines fully connected layers for classification and convolutional layers for feature extraction, is ideally suited for capturing the underlying patterns in the training dataset, as evidenced by the excellent training accuracy. The use of batch normalization and dropout layers contributes to this high performance by normalizing activations, speeding up training, and preventing overfitting during the training phase. However, the validation accuracy of 87.24% and the validation loss of 0.0976 reveal a discrepancy between the model’s performance on the training data and the unseen validation data, as presented in Figure 6. This gap suggests that the model might be overfitting to the training data despite the regularization techniques employed, such as dropout and batch normalization.

When a model learns information and noise from training data that do not transfer to fresh, unobserved data, this is known as overfitting. The relatively lower validation accuracy compared to the training accuracy indicates that while the model performs excellently on the data it was trained on, it does not perform as well on new data, highlighting a potential area for improvement in the model’s ability to generalize. To address this overfitting, further steps could include increasing the dropout rate, adding more data augmentation techniques, or employing early stopping during training. Additionally, gathering more diverse training data or using more sophisticated regularization methods might help in closing the performance gap between training and validation.

**Figure 7:**
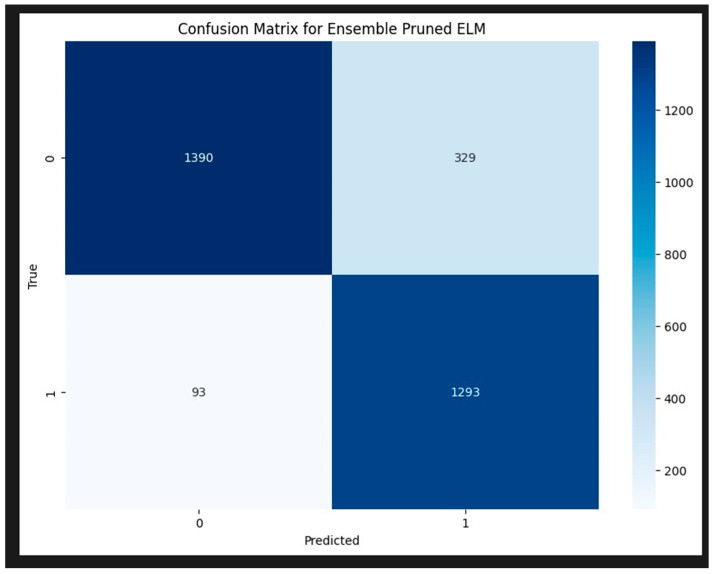
Confusion matrix for HCPELM model.

Despite these challenges, the overall performance of the model is strong, and with some fine-tuning, it has the potential to achieve even better generalization on unseen data. Thus, future research should continue to refines the pruned ELM model to overcome overfitting and achieve better generalization. The confusion matrix for the HCPELM classifier shown in Figure 7 provides important insights into its performance in predicting cancer cases. The HCPELM model shows a strong performance in both sensitivity and specificity. With 1293 true positive cases, the model demonstrates a high ability to correctly identify patients with cancer, which is crucial for timely and effective treatment. The relatively low number of false negatives (93) indicates that the model rarely misses actual cancer cases, reducing the risk of undiagnosed conditions.

Moreover, the model’s ability to correctly classify 1390 true negatives reflects its strength in identifying non-cancerous cases, minimizing unnecessary anxiety and medical procedures for those individuals. However, the presence of 329 false positives suggests that there are some instances where the model incorrectly signals cancer, leading to potential false alarms and additional diagnostic follow-ups. Overall, the HCPELM classifier exhibits a balanced performance with high true-positive and true-negative rates, indicating effective detection of both cancerous and non-cancerous cases. The relatively low false-negative rate is particularly reassuring in a medical context, as it means most cancer cases are correctly identified. While the false-positive rate is moderate, it can be further addressed by refining the model or incorporating additional data and techniques to reduce these instances. This balanced approach enhances the model’s reliability and potential utility in clinical diagnostics.

**Figure 8:**
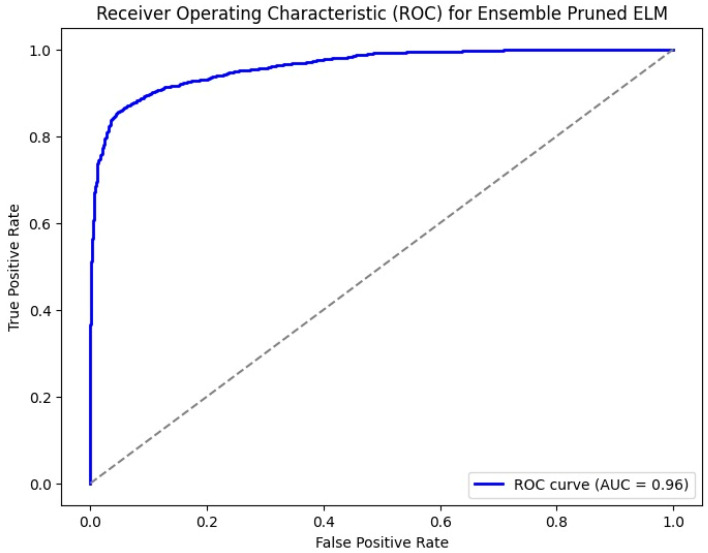
ROC for HCPELM model.

The ROC curve shown in Figure 8 for an ensemble pruned extreme learning machine (ELM) illustrates the classifier’s performance with the true positive rate (sensitivity) on the *y*-axis and the false positive rate on the *x*-axis. The classifier’s strong ability to discriminate between positive and negative classes is demonstrated by the curve’s closeness to the upper left corner, which denotes high sensitivity and specificity. The area under the curve (AUC) is 0.96, signifying that the classifier has a 96% chance of correctly ranking a randomly chosen positive instance higher than a randomly chosen negative one. This high AUC value reflects the classifier’s excellent discriminative power, confirming its effectiveness in accurately identifying true positives while minimizing false positives.

**Figure 9:**
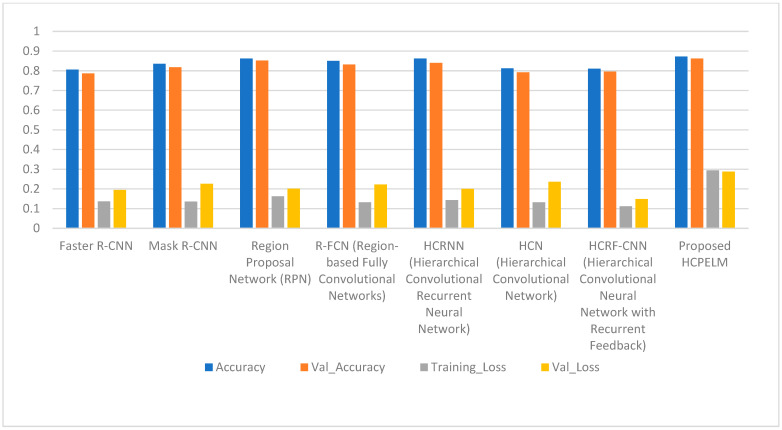
Comparison of proposed HCPELM and other models.

Table 2 shows the obtained results of the pre-trained models and proposed model. Region-based convolutional neural network (RCNN) architectures such as Faster R-CNN and Mask R-CNN demonstrate respectable accuracies of around 80% to 83%, indicating their capability in detecting breast cancer from mammogram images. However, the region proposal network (RPN) stands out with the highest accuracy of 87.25%, highlighting its effectiveness in identifying regions of interest within the images. While RPN excels in accuracy, it is worth noting that Mask R-CNN achieves a slightly higher validation accuracy than Faster R-CNN, albeit with a trade-off of higher validation loss. Hierarchical convolutional neural network (HCNN) models like HCRNN and HCN exhibit accuracies ranging from 81% to 86%, showing competitive performance comparable to RCNN architectures. Among HCNN models, HCRNN stands out for its balanced performance in accuracy and loss metrics. Additionally, the proposed HCPELM model achieves a comparable accuracy of 87.24%, showcasing the potential of hybrid architectures in breast cancer detection tasks. Overall, these findings underscore the importance of selecting appropriate architectures tailored to the complexities of mammogram image analysis, where models like RPN and HCPELM exhibit promising performance metrics. The diagrammatic representation is shown in Figure 9.

### 4.2. Hypothesis Testing and Its Results

To compare the performance of the HCPELM model and the CNN model, we utilized McNemar’s test, a statistical method suitable for comparing paired binary outcomes. The hypotheses for the test are formulated as follows:

- Null Hypothesis (H0): There is no significant difference between the performance of the HCPELM model and the CNN model.
- Alternative Hypothesis (H1): There is a significant difference between the performance of the HCPELM model and the CNN model.

The McNemar’s test was conducted to compare the predictions of the two classifiers on the same dataset. The test results are shown in Table 3.

**Figure 10:**
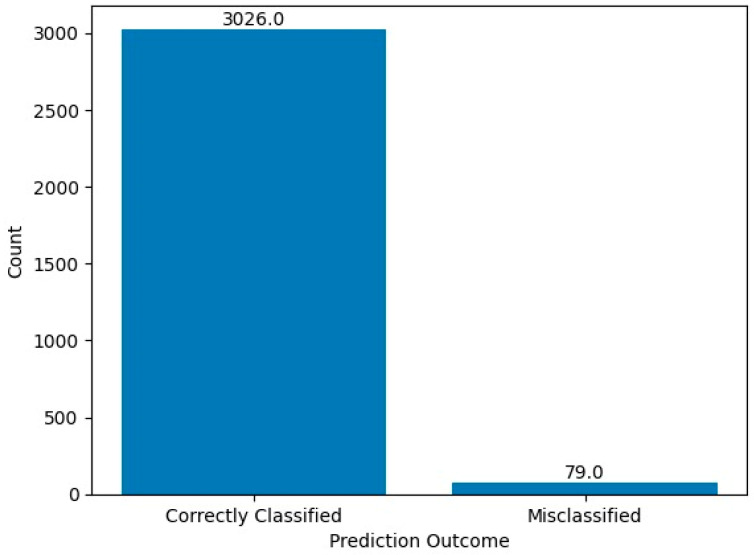
McNemar test results.

The chi-square statistic is 49.192 and the *p*-value is 2.320767726142345 × 10^−12^ (approximately 0.000000000002). Since the *p*-value is significantly less than the commonly used significance level of 0.05, we reject the null hypothesis. This indicates that there is a statistically significant difference between the performance of the HCPELM model and the CNN model. The results show that the HCPELM model significantly outperforms the CNN model. The contingency table, presented in Figure 10, illustrates the correctly and incorrectly predicted values for both models. From this figure, it is evident that the HCPELM model has a higher number of correctly classified instances and a negligible number of incorrectly classified instances, demonstrating the model’s superior accuracy. The McNemar’s test results provide strong evidence that the HCPELM model offers a substantial improvement in performance over the CNN model.

By demonstrating a statistically significant difference, McNemar’s test proves that the HCPELM model is more effective in breast cancer detection and classification compared to the traditional CNN model. This finding is crucial as it highlights the potential of the HCPELM model in providing more accurate diagnostic support for healthcare practitioners.

## 5. Conclusions

The research presented in this paper highlights the development and evaluation of a CAD system that combines convolutional neural networks (CNN) with a pruned ensembled extreme learning machine (HCPELM) to enhance the detection, segmentation, feature extraction, and classification of breast cancer from mammographic images. The hybrid model leverages the strengths of both CNNs and ELMs to improve diagnostic accuracy and reliability. Improved feature extraction and classification are the main findings of this research study. By combining convolutional layers for spatial feature extraction and fully connected layers for non-linear combination and classification, the HCPELM model successfully captures crucial image features like edges and textures. This hybrid approach facilitates the extraction of more meaningful and discriminative features, enhancing the overall classification performance. The use of the rectified linear unit (ReLU) activation function contributed to more efficient data processing and improved analytical capabilities, leading to better model performance in detecting and classifying breast cancer. In artifact and pectoral muscle removal, the pre-processing steps, including artifact and pectoral muscle removal, ensured cleaner and more relevant input data for the model, which is crucial for accurate analysis and classification. In transfer learning and parameter reduction, the application of transfer learning by freezing certain layers and modifying the architecture to reduce parameters proved effective in simplifying the model and enhancing its generalization capabilities. The HCPELM classifier demonstrated superior performance compared to traditional deep learning models, achieving a breast image recognition accuracy of 86% on the MIAS database. This significant improvement underscores the potential of the hybrid model in early detection and diagnosis of breast cancer. The high accuracy and reliability of the HCPELM model makes it a valuable tool for healthcare practitioners. As breast cancer is one of the primary causes of death for women globally, this model’s capacity to support early detection and precise diagnosis may enhance patient outcomes and survival rates. Future work could explore further refinements and adaptations of this hybrid model, as well as its application to other medical imaging tasks to enhance diagnostic processes across various medical domains.

## Figures and Tables

**Table 1:** Breast cancer datasets.

Dataset	Size	Classes/Targets	Format	Type	Author/Repository, Year
MIAS	322	2	pgm	Mammography	Suckling, J. et al. [12]
DDSM INbreast	55,890	410	npy XML	Mammography	Scuccimarra [13] Moreira et al. [14]
Breast Cancer Wisconsin	568	3	csv	Mammography	Dua, D. and Graff, C. [15]
BreakHis	7909	2	png	Histology	Bukun [16]
BACH/ICIAR 2018	400	4	tiff	Histology	G.Aresta [17]

**Table 2:** Comparison of proposed model with other hybrid CNN models.

Models	Accuracy	Val Accuracy	Training Loss	Val_Loss
Faster R-CNN	0.8056	0.7869	0.1366	0.1947
Mask R-CNN	0.8352	0.8182	0.1354	0.2263
Region Proposal Network (RPN)	0.8725	0.8525	0.1625	0.2012
R-FCN (Region-based Fully Convolutional Networks)	0.8506	0.8323	0.1322	0.2221
HCRNN (Hierarchical Convolutional Recurrent Neural Network)	0.8619	0.8403	0.1426	0.1998
HCN (Hierarchical Convolutional Network)	0.8127	0.7926	0.1323	0.2365
HCRF-CNN (Hierarchical Convolutional Neural Network with Recurrent Feedback)	0.8102	0.7956	0.1112	0.1489
Proposed HCPELM	0.8724	0.8621	0.2944	0.2876

**Table 3:** McNemar test results.

Statistic	Value
Chi-square statistic	49.1921568627451
*p*-value	2.320767726142345 × 10^−12^ or 0.000000000002

## Data Availability

The data presented in this study are available on request from the corresponding author.
